# Spender’s Chalk Ointment in Ulcers of the Leg

**Published:** 1856-12

**Authors:** 


### *Spender's Chalk Ointment in Ulcers of the Leg.*

Dr. Patterson has collected 125 cases of chronic non-specific ulcers of the leg, in which, under this mode of treatment, the cure has been rapid and complete. The following formula he prefers: ℞. Cretæ preparatæ, lbiv.; adipis suilli, lbi.; olei olivæ, oz.iis. Having heated the oil and lard, add gradually the chalk, finely powdered.

The ointment and a bandage being once applied, it is left until the cicatrix forms and becomes firm.—*Edinburgh Medical Journal.*

